# Intrauterine Contraceptive Device Translocation Leading to Right Anteromedial Ovarian Surface Impingement and Laparoscopic Retrieval: A Case Report and Literature Review

**DOI:** 10.1002/ccr3.70061

**Published:** 2025-01-06

**Authors:** Adeel Anwaar, Riyan Imtiaz Karamat, Mikail Khanzada, Aymar Akilimali, Minahil Aamir, Mirza Ammar Arshad, Ihtisham Haider Bhatti, Saad Rashid, Taimur Sulaiman Kayani, Sadia Ansar, Shifa Batool, Abdullah Saif Khan, Ajeet Singh

**Affiliations:** ^1^ Research Associate at Urology Suite Midcity Hospital Lahore Pakistan; ^2^ Department of Medicine Rahbar Medical and Dental College Lahore Pakistan; ^3^ Department of Medicine Lahore Medical and Dental College Lahore Pakistan; ^4^ Department of Research Medical Research Circle (MedReC) Goma Democratic Republic of the Congo; ^5^ Department of Medicine Dow Medical College Karachi Pakistan; ^6^ Department of Medicine Aga Khan University Karachi Pakistan; ^7^ Department of Medicine Rawal Institute of Health Sciences Islamabad Pakistan; ^8^ Department of Medicine Hamdard College of Medicine and Dentistry Karachi Pakistan

**Keywords:** case report, devices, intrauterine contraceptive, laparoscopy, uterine perforation

## Abstract

We report a rare case of a 29‐year old woman presenting with abdominal pain, whose initial examination failed to identify intrauterine contraceptive device (IUCD) threads. IUCD migration was confirmed by CT scan and subsequent single‐port laparoscopic retrieval alleviated her symptoms.

## Graphical abstract

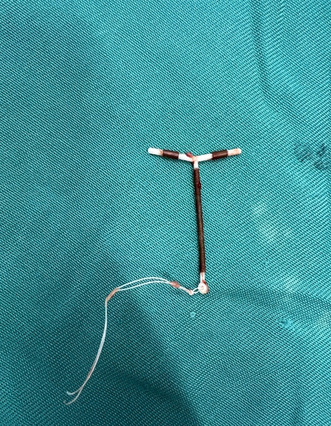

**Abbreviations**
CT‐Pelvis: computed tomography scan of pelvis
CT scan: computed tomography scan
IUD: intrauterine contraceptive device
LARC: long‐acting reversible contraception
USG‐ ABD: ultrasound of abdomen
USG‐Pelvis: ultrasound of pelvis
USG‐TV: transvaginal
UTI: urinary tract infection

## 1 Introduction

When considering contraceptive options, intrauterine contraceptive devices (IUDs) stand out as one of the most effective choices of long‐acting reversible contraception (LARC) currently available [1]. IUDs are widely regarded as the most reliable method of pregnancy prevention, they come with a range of potential side effects some common ones being perforation, infections, heavy bleeding, or risk of expulsion [2]. IUD migration is a notable adverse effect, potentially leading to complications such as pelvic pain, dyspareunia, gastrointestinal perforation, and expulsion, among others. The rare but serious risk of partial or total perforation, which carries significant therapeutic implications, is especially concerning. For levonorgestrel‐releasing IUDs, the rates of uterine perforation vary between 0.3 and 2.6 per 1000 insertions, while copper IUDs have similar rates ranging from 0.3 to 2.6 per 1000 [3, 4, 5, 6]. Risk factors for IUD migration include nulliparity, uterine scars, placement during breastfeeding, or insufficient experience of the healthcare provider [7]. Furthermore, the possibility of an ectopic IUD migrating from the uterus to unexpected locations within the body, such as the appendix, rectum, or bladder, highlights the risks associated with their use [8, 9, 10]. Investigations to locate misplaced IUCDs generally include ultrasound, hysteroscopy, and CT scans with ultrasound generally being the commonly used first‐line investigation [11]. Treatment requires surgical removal either by exploratory laparotomy or laparoscopic removal of the IUCD [12].

We present a compelling case that details the rarity, diagnostic process, and management approach for an uncommon occurrence of a translocated IUD embedded within the right ovary.

## 2 Clinical Presentation

### 2.1 Case History/Examination

A 29‐year‐old mother of one presented to our emergency department with complaints of lower abdominal pain persisting for the past few days. There was no reported history of urinary tract infection (UTI), abnormal vaginal bleeding, or kidney stones. On physical examination, the abdomen was tender on palpation. The patient has been married since 2019 and had a Copper‐T 380A IUD inserted 6 months prior with no bleeding or discomfort reported at the time of insertion. During the in‐clinic evaluation, we were unable to visually locate the IUD threads despite conducting a thorough bimanual vaginal and speculum examination.

### 2.2 Investigations

**FIGURE 1:**
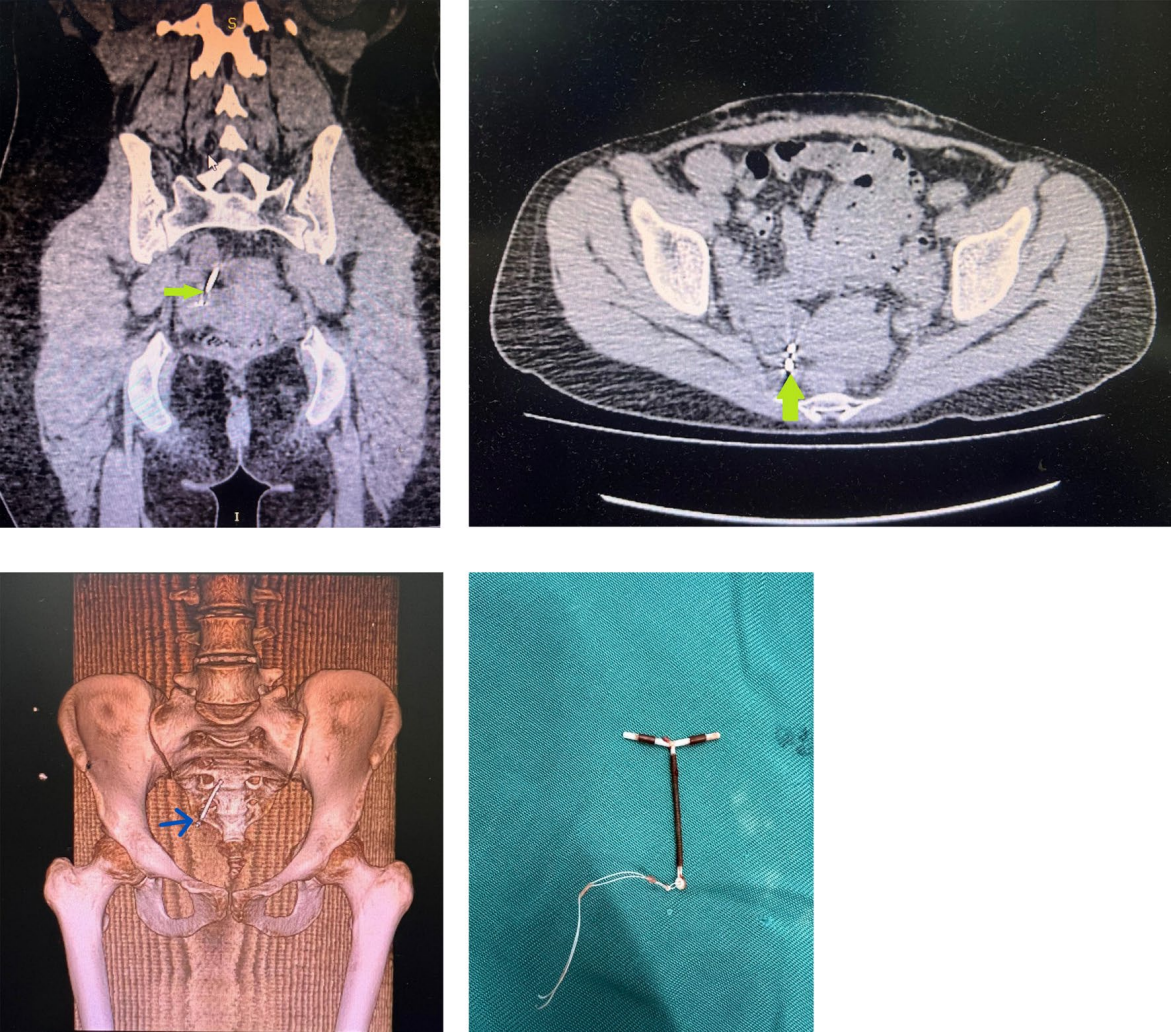
(a) CT—Pelvis coronal displaying a transmigrated T‐shaped IUD with long arm directed superomedially and short T‐arm potentially impinging onto a right ovarian surface. (b) CT—Pelvis axial view showing IUD in close proximity to right ovary. (c) CT 3D view of the IUD. (d) Showing the actual translocated IUD postlaparoscopic retrieval.

Initial ultrasound of the kidneys, ureters, and bladder was found to be inconclusive. A subsequent transvaginal ultrasound (TV‐USG) revealed an empty uterus with no trace of the intrauterine device (IUD). This prompted further investigation through a computed tomography scan of the pelvis (CT‐pelvis). The CT scan revealed the displaced IUD located in the pelvis, ex situ, positioned to the right of the uterus. It was situated anterior to the anteromedial aspect of the right ovary, with the long limb of the IUD oriented upwards and the short limb appearing to impinge upon the anteromedial ovarian surface. Importantly, there were no indications that the IUD was penetrating any bowel loops. No free fluid or collections were noted in the pelvic cavity. The 3D CT scan showed no signs of any deposits or bony destruction associated with the presence of this foreign body in the adnexal region (Figure 1a–d).

### 2.3 Treatment Intervention

**FIGURE 2:**
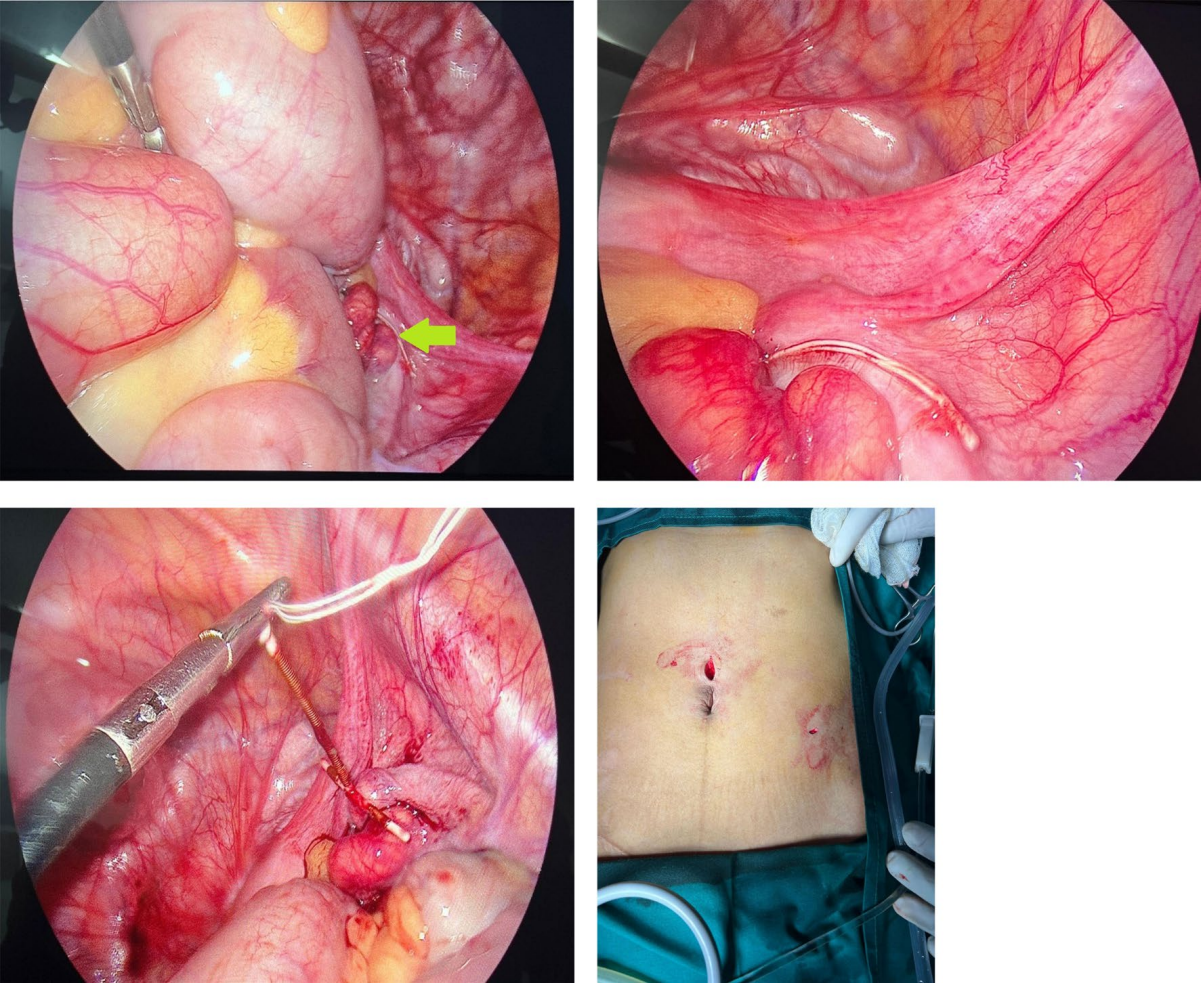
(a) IUD thread spotted next to right ovary. (b) Close‐up view of IUD thread. (c) IUD thread held with laparoscopic grasper in process of removal via second port. (d) Postoperative view of laparoscopic ports.

All treatment options were clearly explained to the patient, prioritizing cosmetic considerations that were important to her. After obtaining informed consent, we decided to proceed with a laparoscopic removal of the IUD, confident in both the ease of access and the localization of the IUD within the abdomen. A 10‐mm supraumbilical port was inserted using Hasson's technique, and pneumoperitoneum was established with carbon dioxide gas. Subsequently, a 5 mm port was placed in the left lower quadrant. A grasper was employed to locate the IUD in the right pelvic cavity, where its thread was ultimately identified. The IUD, embedded in the right ovary, was gently extracted with an atraumatic grasper and successfully removed through the 5 mm port. Suturing was deemed unnecessary as no bleeding was detected at the removal site. No signs of intra‐abdominal iatrogenic injury were noted prior to the withdrawal of the laparoscope. Additionally, there were no indications of uterine perforation during the laparoscopic examination. A visual representation of key steps in the procedure and the retrieved IUD is provided below (Figure 2a–d).

### 2.4 Postoperative Outcome and Follow‐Up

The patient was discharged the following morning with a recommendation for 1 week of bed rest. At the scheduled 3‐week postoperative follow‐up, she exhibited complete resolution of her initial complaint. The patient was referred to a gynecologist for discussion of subsequent contraceptive methods. On follow‐up, over the counter oral contraceptives were prescribed for future contraception needs.

## 3 Discussion

IUD‐related perforations are a rare complication most commonly encountered at the time of implantation [13]. Later perforations typically occur due to slow erosion of the uterine wall. Early migrations, which typically occur within the first few months after insertion, can result from these initial placement issues or physiological factors such as uterine contractions, often manifesting as irregular bleeding, cramping, or a sensation of expulsion. In contrast, late migrations develop over a longer period, often linked to gradual uterine changes or conditions like fibroids, leading to symptoms such as chronic pelvic pain, signs of infection, or even complications like perforation. Our findings align with existing literature but also highlight how specific manifestations in our case provide insights into the timing and clinical presentation of IUD migration, emphasizing the importance of ongoing monitoring and individualized patient assessment [14, 15]. The anterior cervical fornix is the most frequent site of perforation; in this case, the patient's IUD was found perforating the anterior‐median portion of the cervix [16]. Case reports have documented IUD migration to various sites, including the bowel, bladder, parametrium, uterus, cervix, mesentery, appendix, omentum, space of Retzius, and both anterior and posterior cul‐de‐sacs [11].

The occurrence of ovarian transmigration of an IUD is rare, with only a few other cases reported, making our case particularly unusual. Approximately 15% of perforations lead to complications affecting other organs, often resulting in peritonitis after bowel segments are perforated [12]. Ultrasound remains the preferred imaging technique for detecting perforated IUDs, as they appear as echogenic structures. However, in this instance, a CT scan of the pelvis was necessary for a definitive diagnosis [16]. CT scans also offer superior accuracy in locating perforated IUDs compared to X‐rays. Current medical guidelines indicate that perforated intrauterine devices (IUDs) should only be removed if the patient experiences symptoms or if there is a potential risk of adhesions, additional perforations, or other complications [17, 18]. In our patient's case, given her moderate pain, we decided to proceed with the removal of the device. This case involves a woman with right ovarian transmigration of an IUD and represents one of the few instances of successful laparoscopic removal [6, 7, 8].

Although laparotomy has traditionally been the preferred surgical approach in most cases, laparoscopic removal provides several advantages, including enhanced safety, quicker recovery times, and more aesthetically pleasing scars. However, laparoscopic removal should be avoided if the IUD is deeply embedded in surrounding tissue or has caused significant organ damage [19, 20]. Mosley et al. noted that 22.5% of planned laparoscopies required conversion to laparotomy in patients whose IUDs had migrated into the peritoneal cavity [21]. Similarly, Gill et al. reported a 21% success rate for laparoscopic removal in cases involving bowel perforation [22]. Nevertheless, for uncomplicated situations, attempting laparoscopic removal is recommended as the first‐line treatment for both symptomatic and asymptomatic patients.

Our case presented with mild symptoms of intrauterine device (IUD) perforation, particularly when compared to other cases involving ovarian transmigration. Common symptoms associated with IUD perforation and migration to the ovary included abdominal pain, vaginal bleeding, and dysmenorrhea. In our literature review of nine cases of ovarian transmigration, four—including our case—were resolved using laparoscopy [6, 8, 9], four required laparotomies, and two necessitated salpingo‐oophorectomy. Notably, the right ovary was involved in six out of the nine cases, while the left ovary was affected only three times. Ultrasound was the preferred method of investigation in the majority of cases, with only three of the eight cases, excluding ours, requiring further evaluation via CT scan [1, 2, 3, 4, 5, 6, 8, 9]. Table 1 describes the comparison of our case report with previous studies [1, 3, 4, 5, 6, 8, 9, 10].

**TABLE 1:** Literature review for case reports having IUD translocation to an ovary.

Study	Presenting complaints	Past medical history	Location of IUD perforation	Imaging investigations employed	Retrieval technique
Our case (2024)	Lower abdominal pain for the past few days	Intrauterine contraceptive device (IUD) inserted for the past 6 months	Right anteromedial ovarian surface	USG‐TV USG‐KUB CT‐pelvis	Laparoscopy
Lafraia et al. (2023) [1]	Intermittent pain in the right iliac fossa associated with minor vaginal bleeding for the last 30 days	No past history	Right ovary	USG‐TV	Right salpingo‐oophorectomy
Diaouga HS et al. (2022) [3]	Three cesarean births Violent pelvic pain felt at the time of the insertion of the IUD	Intense pelvic pain evolving for 24 h before her admission	Right ovary	USG‐pelvis	Laparotomy
Kaushik A et al. (2020) [4]	Severe lower abdominal pain since 10 days Vomiting since 4 days	Postpartum IUD insertion 10 years back	Left ovary	TAS USG‐TV	Laparotomy
An Y et al. (2020) [5]	Vaginal bleeding Lower abdominal pain persistent cough for 3 months Pneumonia	First delivery 37 years ago IUD insertion one and a half year Pregnancy 3 months later to the IUD insertion	Right adnexa	USG‐ABD CT scan Hysteroscopy	Hysterectomy Bilateral salpingo‐oophorectomy Omentectomy Laparotomy
Aminu MB et al. (2019) [6]	Lower abdominal pain Inability to feel the string of copper IUD that was inserted at 6 weeks postpartum	Pain and bleeding following the insertion Child Birth 3 months prior to presentation to set of twins with no puerperal complications	Left ovary	USG‐ABD	Laparotomy
Rovati et al. (2016) [8]	Severe dysmenorrhea, single episode of lower abdominal pain for 24 h, 2 weeks earlier Pain on the right side	No past history	Right ovary	USG‐TV X‐ray CT scan	Laparoscopy
Verma et al. (2009) [9]	Menorrhagia Severe dysmenorrhea Dyspareunia Constant and nonradiating pain mostly on the right side worsening over the last 6 months Complaint of lower abdominal pain for 3 years	Appendectomy	Right ovary	USG‐pelvis	Laparoscopy
Ozdemir H et al. (2004) [10]	Lower Abdominal Pain	History of 2 IUD insertions, Pregnancy despite IUD insertion, IUD threads not found.	Left ovary	USG‐TV CT‐pelvis	Laparoscopy

## 4 Conclusion

Clinicians should always consider IUCD translocation or uterine perforation as potential differentials in female patients presenting with acute lower abdominal pain. An immediate vaginal speculum examination and ultrasound study should be ordered in such cases. We strongly recommend that only well‐trained medical professionals perform IUD insertions to minimize complications, including IUCD transmigration and potential damage to abdominal viscera or intraperitoneal pelvic organs.

## Author Contributions

**Adeel Anwaar:** data curation, writing – review and editing. **Riyan Imtiaz Karamat:** conceptualization, project administration, supervision, visualization, writing – original draft. **Mikail Khanzada:** writing – original draft. **Aymar Akilimali:** methodology, project administration, supervision, writing – review and editing. **Minahil Aamir:** writing – original draft. **Mirza Ammar Arshad:** methodology, software, writing – original draft. **Ihtisham Haider Bhatti:** validation, writing – review and editing. **Saad Rashid:** methodology, validation, writing – review and editing. **Taimur Sulaiman Kayani:** conceptualization, methodology, writing – review and editing. **Sadia Ansar:** supervision, validation, writing – review and editing. **Shifa Batool:** formal analysis, writing – review and editing. **Abdullah Saif Khan:** writing – review and editing. **Ajeet Singh:** supervision, validation, writing – review and editing.

## Ethics Statement

The authors have nothing to report.

## Consent

A written informed consent was obtained from the patient based on the journal's policies.

## Conflicts of Interest

The authors declare no conflicts of interest.

## Data Availability

The data that support the findings of this study are available from the corresponding author upon reasonable request.

## References

[1] F. M. Lafraia , A. L. D. Barbosa , L. A. Zorzanelli , et al., “Ovary Transmigration of a Levonorgestrel‐Releasing Intrauterine Device and Ectopic Pregnancy: A Case Report,” Human Reproduction Archives 38 (2023): e000521.

[2] T. C. Jatlaoui , H. E. M. Riley , and K. M. Curtis , “The Safety of Intrauterine Devices Among Young Women: A Systematic Review,” Contraception 95, no. 1 (2017): 17–39, 10.1016/j.contraception.2016.10.006. 27771475 PMC6511984

[3] H. S. Diaouga , M. C. Yacouba , H. Soumaila , M. R. Garba , N. Idi , and M. Nayama , “Intraovarian Migration of the Intrauterine Device; Complicated by Haemorrhagic Ovarian Cyst,” International Journal of Reproduction, Contraception, Obstetrics and Gynecology 11, no. 8 (2022): 2260–2263.

[4] A. Kaushik , D. S. Rajpurohit , K. Chaturvedy , et al., “Partial Uterine Perforation and Ovarian Embedment of Misplaced Intrauterine Device: A Case Report,” International Journal of Reproduction, Contraception, Obstetrics and Gynecology 9, no. 12 (2020): 5114–5117.

[5] Y. An , C. Liu , F. Mao , G. Yang , and G. Mao , “Intrauterine Device Found in an Ovarian Tumor: A Case Report,” Medicine 99, no. 42 (2020): e22825, 10.1097/MD.0000000000022825. 33080762 PMC7571936

[6] M. B. Aminu , L. M. Dattijo , and M. S. Adamu , “Ovarian Penetration by Copper Intrauterine Device: A Rare Phenomenon,” Saudi Journal of Health Sciences 7 (2018): 183–185.

[7] V. Verstraeten , K. Vossaert , and T. Van den Bosch , “Migration of Intra‐Uterine Devices,” Open Access Journal of Contraception 12, no. 15 (2024): 41–47, 10.2147/OAJC.S458156. PMC10944303 38495451

[8] M. Rovati , F. Raveglia , A. Baisi , M. De Simone , and U. Cioffi , “Ovarian Transmigration of Intrauterine Device,” Journal of Obstetrics and Gynaecology Research 42, no. 12 (2016): 1889–1890, 10.1111/jog.13120. 27991745

[9] U. Verma and N. Verma , “Ovarian Embedding of a Transmigrated Intrauterine Device: A Case Report and Literature Review,” Archives of Gynecology and Obstetrics 280, no. 2 (2009): 275–278. 19096860 10.1007/s00404-008-0882-2

[10] H. Ozdemir , K. Mahmutyazicioğlu , H. A. Tanriverdi , S. Gündoğdu , A. Savranlar , and T. Ozer , “Migration of an Intrauterine Contraceptive Device to the Ovary,” Journal of Clinical Ultrasound 32, no. 2 (2004): 91–94, 10.1002/jcu.10228. 14750141

[11] K. Andersson , E. Ryde‐Blomqvist , K. Lindell , V. Odlind , and I. Milsom , “Perforations With Intrauterine Devices. Report From a Swedish Survey,” Contraception 57, no. 4 (1998): 251–255, 10.1016/s0010-7824(98)00029-8. 9649917

[12] D. Zakin , W. Z. Stern , and R. Rosenblatt , “Complete and Partial Uterine Perforation and Embedding Following Insertion of Intrauterine Devices. I. Classification, Complications, Mechanism, Incidence, and Missing String,” Obstetrical & Gynecological Survey 36, no. 7 (1981): 335–353, 10.1097/00006254-198107000-00001 PMID: 7029368. 7029368

[13] S. E. Stephen , “The Intrauterine Device and the Intrauterine System,” Best Practice & Research. Clinical Obstetrics & Gynaecology 28, no. 6 (2014): 807–824, 10.1016/j.bpobgyn.2014.05.004. 24947600

[14] X. Sun , M. Xue , X. Deng , Y. Lin , Y. Tan , and X. Wei , “Clinical Characteristic and Intraoperative Findings of Uterine Perforation Patients in Using of Intrauterine Devices (IUDs),” Gynecological Surgery 15, no. 1 (2018): 3, 10.1186/s10397-017-1032-2. 29386988 PMC5770510

[15] K. Heinemann , S. Reed , S. Moehner , and T. D. Minh , “Risk of Uterine Perforation With Levonorgestrel‐Releasing and Copper Intrauterine Devices in the European Active Surveillance Study on Intrauterine Devices,” Contraception 91, no. 4 (2015): 274–279. 25601352 10.1016/j.contraception.2015.01.007

[16] P. Peitsidis , C. Ekizoglou , D. Spiliopoulos , S. Zervoudis , K. Kalmantis , and P. Tsikouras , “Perforation of the Cervix by the Strings of an Intrauterine Device (IUD): A Novel Case and Systematic Review of the Literature,” Maedica 17, no. 3 (2022): 699–705, 10.26574/maedica.2022.17.3.699. 36540590 PMC9720661

[17] D. K. Turok , S. E. Gurtcheff , K. Gibson , E. Handley , S. Simonsen , and P. A. Murphy , “Operative Management of Intrauterine Device Complications: A Case Series Report,” Contraception 82, no. 4 (2010): 354–357, 10.1016/j.contraception.2010.04.152. 20851229

[18] J. Kaislasuo , S. Suhonen , M. Gissler , P. Lähteenmäki , and O. Heikinheimo , “Uterine Perforation Caused by Intrauterine Devices: Clinical Course and Treatment,” Human Reproduction 28, no. 6 (2013): 1546–1551, 10.1093/humrep/det074. 23526304

[19] F. Tabatabaei and M. Masoumzadeh , “Dislocated Intrauterine Devices: Clinical Presentations, Diagnosis and Management,” European Journal of Contraception & Reproductive Health Care 26, no. 2 (2021): 160–166, 10.1080/13625187.2021.1874337. 33555216

[20] D. Sahu , K. K. Sao , and S. S. Dubey , “Laparoscopic Retrieval of a Displaced Intrauterine Device Presenting as Umbilicus Sinus,” World Journal of Laparoscopic Surgery 13, no. 2 (2020): 87–89.

[21] F. R. Mosley , N. Shahi , and M. A. Kurer , “Elective Surgical Removal of Migrated Intrauterine Contraceptive Devices From Within the Peritoneal Cavity: A Comparison Between Open and Laparoscopic Removal,” Journal of the Society of Laparoendoscopic Surgeons 16, no. 2 (2012): 236–241, 10.4293/108680812x13427982377265. 23477171 PMC3481248

[22] R. S. Gill , D. Mok , M. Hudson , X. Shi , D. W. Birch , and S. Karmali , “Laparoscopic Removal of an Intra‐Abdominal Intrauterine Device: Case and Systematic Review,” Contraception 85, no. 1 (2012): 15–18, 10.1016/j.contraception.2011.04.015. 22067801

